# Gender Differences in the Atherosclerosis Profile by Coronary CTA in Coronary Artery Calcium Score Zero Patients

**DOI:** 10.3390/jcm10061220

**Published:** 2021-03-15

**Authors:** Thomas Senoner, Fabian Plank, Christoph Beyer, Christian Langer, Katharina Birkl, Fabian Steinkohl, Gerlig Widmann, Fabian Barbieri, Agne Adukauskaite, Guy Friedrich, Axel Bauer, Wolfgang Dichtl, Gudrun M. Feuchtner

**Affiliations:** 1 Department of Internal Medicine III—Cardiology and Angiology, Innsbruck Medical University, 6020 Innsbruck, Austria; fabian.plank@i-med.ac.at (F.P.); fabian.barbieri@i-med.ac.at (F.B.); agne.adukauskaite@i-med.ac.at (A.A.); guy.friedrich@i-med.ac.at (G.F.); axel.bauer@i-med.ac.at (A.B.); wolfgang.dichtl@i-med.ac.at (W.D.); 2 Department of Radiology, Innsbruck Medical University, 6020 Innsbruck, Austria; christoph.beyer@student.i-med.ac.at (C.B.); christian.langer@i-med.ac.at (C.L.); katharina.birkl@i-med.ac.at (K.B.); fabian.steinkohl@i-med.ac.at (F.S.); gerlig.widmann@i-med.ac.at (G.W.); 3 Department of Radiology, St. Vinzenz Hospital, 6511 Zams, Austria

**Keywords:** coronary artery disease, computed tomography angiography, coronary artery calcium score, high-risk plaque criteria, gender differences

## Abstract

*Background*: The coronary artery calcium score (CACS) is a powerful tool for cardiovascular risk stratification. Coronary computed tomography angiography (CTA) allows for a more distinct analysis of atherosclerosis. The aim of the study was to assess gender differences in the atherosclerosis profile of CTA in patients with a CACS of zero. *Methods*: A total of 1451 low- to intermediate-risk patients (53 ± 11 years; 51% females) with CACS <1.0 Agatston units (AU) who underwent CTA and CACS were included. Males and females were 1:1 propensity score-matched. CTA was evaluated for stenosis severity (Coronary Artery Disease – Reporting and Data System (CAD-RADS) 0–5: minimal <25%, mild 25–49%, moderate 50–69%, severe ≥70%), mixed-plaque burden (G-score), and high-risk plaque (HRP) criteria (low-attenuation plaque, spotty calcification, napkin-ring sign, and positive remodeling). All-cause mortality, cardiovascular mortality, and major cardiovascular events (MACEs) were collected. *Results*: Among the patients, 88.8% had a CACS of 0 and 11.2% had an ultralow CACS of 0.1–0.9 AU. More males than females (32.1% vs. 20.3%; *p* < 0.001) with a CACS of 0 had atherosclerosis, while, among those with an ultralow CACS, there was no difference (88% vs. 87.1%). Nonobstructive CAD (25.9% vs. 16.2%; *p* < 0.001), total plaque burden (2.2 vs. 1.4; *p* < 0.001), and HRP were found more often in males (*p* < 0.001). After a follow-up of mean 6.6 ± 4.2 years, all-cause mortality was higher in females (3.5% vs. 1.8%, *p* = 0.023). Cardiovascular mortality and MACEs were low (0.2% vs. 0%; *p* = 0.947 and 0.3% vs. 0.6%; *p* = 0.790) for males vs. females, respectively. Females were more often symptomatic for chest pain (70% vs. 61.6%; *p* = 0.004). (4) *Conclusions*: In patients with a CACS of 0, males had a higher prevalence of atherosclerosis, a higher noncalcified plaque burden, and more HRP criteria. Nonetheless, females had a worse long–term outcome and were more frequently symptomatic.

## 1. Introduction

Coronary artery disease (CAD) is the major cause of death in developed countries, with a higher prevalence in males than females [1]. Cardiovascular disease develops 7 to 10 years later in females [1]. The long-standing observation that ovarian steroid hormones and, in particular, estrogens are cardioprotective has been refuted by randomized clinical trials of both primary and secondary prevention of atherosclerotic cardiovascular disease (ASCVD) [2,3]. Recent data indicate worse outcomes in women with CAD as compared with men. Women are less likely to be referred for functional testing for ischemia, undergo fewer interventional procedures, and receive less evidence-based medical treatment compared with men [4].

A coronary artery calcium score (CACS) of zero calculated from computed tomography (CT) scanning is advocated for a safe exclusion of CAD, with a very low mortality rate of 0.11% [5] in asymptomatic low- to intermediate-risk individuals after 10 years, as shown in numerous large cohorts [6] (“the power of CACS 0”). Furthermore, the CACS score is a powerful tool for coronary risk stratification; however, due to a lack of contrast agent application, hypodense noncalcified plaque, representing early stages of atherosclerosis, remain undetected.

In contrast to nonenhanced CACS scans, coronary computed tomography angiography (CTA) allows for the evaluation of coronary stenosis severity and plaque burden, as well as for a more detailed analysis of the atherosclerosis profile: quantification of noncalcified low-attenuation plaque (“fibroatheroma”) and high-risk plaque (HRP) criteria, such as low-attenuation plaque (LAP), napkin-ring sign, spotty calcification, and positive remodeling, presenting novel imaging biomarkers for increased cardiovascular risk. High-risk plaque criteria pose the patient to a 59-fold higher risk of major cardiovascular events (MACEs) [7,8,9,10,11]. In particular, the necrotic core plaque (LAP <30 Hounsfield Units (HU)) is one of the most powerful predictors of MACEs.

However, the rate of significant coronary artery disease by CTA in patients with a CACS of zero has shown high variations from 7–32% [12,13] with a trend to a low prevalence of obstructive disease [14,15]. To date there is a lack of data regarding gender differences in the prevalence of high-risk plaque features [8,9,10,11] by CTA.

Furthermore, it has been suggested to classify patients with a CACS <1.0 Agatston units (AU) as “CACS zero” patients [6]. No study yet has analyzed the CAD profile by CTA between males and females in terms of coronary stenosis severity, total plaque burden, and high-risk plaque features, including a quantitative plaque analysis in ultralow CACS (0.1–0.9 AU) patients.

Therefore, the purpose of our study was to assess gender differences in the coronary atherosclerosis profile by coronary CTA in patients with CACS 0, with regard to stenosis severity, total plaque burden, and high-risk plaque criteria, as well as to perform a subanalysis in those with ultralow CACS (0.1–0.9 AU). Furthermore, long-term outcome (all-cause, cardiovascular mortality and MACEs) data were collected.

## 2. Materials and Methods

### 2.1. Study Design and Population

A total of 6439 consecutive patients referred for coronary CTA for clinical indication between November 2005 and December 2018 were entered into our database and screened. Among them, 1451 low- to intermediate-ASCVD-risk (mean ASCVD of 9.225%) patients had a CACS <1.0 AU and were included in the study. The retrospective cohort study was approved by our local institutional review board, and patients’ informed consent forms were waived.

### 2.2. Inclusion Criteria

Patients with unknown CAD and low to intermediate ASCVD risk [16], due to typical or atypical chest pain, or asymptomatic patients with a clinical suspicion of CAD (e.g., pathological resting electrocardiogram (ECG), borderline or nonspecific ECG treadmill, or abnormal myocardial perfusion stress test with equivocal findings). Conventional coronary risk factors according to European Society of Cardiology guidelines were collected: arterial hypertension [17], dyslipidemia [18], positive family history, smoker (current or quit within the last 6 months), and diabetes [19].

### 2.3. Exclusion Criteria

Exclusion criteria were as follows: known CAD, previous percutaneous coronary intervention, coronary artery bypass grafting, previous myocardial infarction, heart valve surgery, severe aortic stenosis, atrial fibrillation, renal dysfunction (serum glomerular filtration rate (GFR) <60 mL/min/1.73 m^2^), pregnancy, and age <21 years. Patients with positive troponin and unstable angina were excluded.

### 2.4. Primary Endpoint Was All-Cause Mortality, Derived from Our National Death Register

Cardiovascular mortality and major cardiovascular event (MACE) data were collected. A MACE was defined as acute coronary syndrome-ST-elevation myocardial infarction (ACS-STEMI), non-STEMI, or ACS by in-hospital documentation and/or by postmortem histology of myocardial infarction [20].

### 2.5. Coronary Computed Tomography Angiography (CTA)

Computed tomography angiography was performed [21] as follows: a non-contrast ECG-gated coronary calcium score (CACS) with standardized scan parameters (detector collimation 64 × 1.5 mm;120 kV) was performed, and the Agatston Score (measured in AU) [22] was calculated.

Coronary CTA was performed either with a 128-slice dual source CTA (Definition FLASH, Siemens, Forchheim, Germany) with a detector collimation of 2 × 64 × 0.6 mm, a *z*-flying spot, and a rotation time of 0.28 s or a 64-slice CTA (Somatom 64, Siemens, Forchheim, Germany) with a detector collimation of 64 × 0.6 mm and a rotation time of 0.33 s. Prospective ECG-triggering was used in regular heart rates <65 bpm (70% of RR interval), retrospective ECG gating in heart rates >65 bpm, and irregular rates.

An iodine contrast agent (Iopromide, Ultravist 370™) was injected intravenously (flow rate 4–6 mL/s + 40 cc saline), triggered into the arterial phase (bolus tracking; 100 HU threshold; ascending aorta). Contrast volume ranged from 65 to 120 cc according to the individual patient characteristics. Axial images were reconstructed with 0.75 mm slice width (increment 0.4/medium-smooth kernel B26f) during the best diastolic and systolic phase.

### 2.6. Coronary CTA Image Analysis

Curved multiplanar reformations (cMPRs) and oblique interactive MPRs using client–server-based three-dimensional (3D) post-processing software (SyngoVia^TM^, Siemens Healthineers, Forchheim, Germany) were generated:

1. Coronary stenosis severity was scored qualitatively according to Coronary Artery Disease – Reporting and Data System (CAD-RADS^TM^) score (0–5) [23] as minimal (1) <25%, mild (2) 25–49.9%, moderate (3) 50–69.9%, severe (4) ≥70%–99%, and 100% on a per coronary segment basis (American Heart Association (AHA)-modified 17-segment classification) [24].
2. Plaque types were characterized semiquantitative as follows: type 1 = calcified, type 2 = mixed (predominantly calcified), 3 = mixed (predominantly noncalcified), 4 = noncalcified per AHA coronary segment. Calcified and noncalcified plaque were defined as hyper- and hypoattenuating lesions (<150 HU) [25], respectively. The total plaque burden (G-score), a per patient measure with greater weighting of noncalcified plaque components, was calculated as previously described [26].
3. High-risk plaque (HRP) analysis:
  - Low-attenuation plaque (LAP) was defined as a hypoattenuating lesion with <150 HU [25]. CT density was screened with the “pixel lens” and the lowest HU recorded [9]. Then, an area ROI (region of interest) of approximately 2 mm^2^ size was placed at the region of lowest density and drawn as large as possible, while sparing areas affected by artefacts or adjacent to calcifications, and the CT attenuation (HU) was quantified. If a patient had multiple lesions, the one with the lowest HU was selected for a patient-based analysis. LAP was subdivided into LAP <90 HU, LAP <60 HU (fibrofatty) (11), and LAP <30 HU (lipid-rich necrotic core) [10].
  - Napkin-ring sign (NRS) was defined as an outer high-density rim with an inner hypodense area [8].
  - Spotty calcification (SC) was defined as a calcification of less than 3 mm size.
  - The remodeling index (RI) was calculated as the ratio of the maximal cross-sectional lumen of the plaque diameter and its closest proximal (or distal, e.g., in case of ostial lesions) normal reference vessel lumen diameter. Positive remodeling was defined as an RI >1.1.

A HRP was identified if a minimum of two out of four criteria were present (according to label “V”, CAD-RADS) [23], while LAP <30 HU (10) was defined as a necrotic core plaque, and LAP < 60 HU (11) was defined as fibrofatty. Both were regarded as “high-risk” plaque criteria. In the case of multiple lesions, all HRPs were quantified, and the number of HRPs per patient was recorded.

CTA image analysis was performed by two independent observers (one observer with SCCT (society of cardiovascular computed tomography) level II training, and one observer with SCCT level III training and 10 years of cardiac CT experience). Consensus reading was obtained. Plaques with image quality limitations such as artefacts (motion blurring, high image noise, beam hardening, or streak artefacts) were excluded from quantitative HRP analysis (approximately 4% of patients).

### 2.7. Outcome and Follow-Up Data Collection

Serial follow-up was performed via phone interview every 3 years after CTA, and hospital chart results were checked regularly until September 2018. Additionally, after 10 years, all patients were entered into the Austrian Mortality Register, a query was obtained, and all entries were verified.

### 2.8. Statistical Analysis

Statistical analysis was performed using SPSS™ software (V24.0, SPSS Inc., Chicago, IL, USA). Quantitative variables are expressed as means ± standard deviation (SD), while categorical variables are expressed as absolute values and percentages.

For assessing gender differences between the two CACS groups, a propensity score matchmaking (PSM) model was calculated. Matching ensures that the distributions of confounding variables are identical (or as close to identical as possible) so as to compare the case group with the control group, thereby reducing selection bias. A binary regression was conducted including age, body-mass index (BMI), and five major risk factors (arterial hypertension, smoking, positive family history, dyslipidemia, and diabetes). Given probabilities were then matched using a 1:1 nearest neighbor matchmaking process without replacement. Matching tolerance was set to 0.01, which resulted in 371 males and females, respectively, divided into 320 males and 328 females in the CACS 0 AU group and 51 males and 43 females in the ultralow CACS group.

Differences in all parametric data between two groups were tested using the independent *t*-test in case of normal distribution or Mann–Whitney U for non-normally distributed and rank-scaled variables (such as total plaque burden (G-score) and CACS score). A Kruskal–Wallis test was used to test for differences in CAD-RADS score between groups. To assess the distribution, the Kolmogorov–Smirnov test and histograms were used. Differences in categorical data were determined with the chi-square or Fisher’s exact test (if *n* < 5 per group).

A two-sided *p*-value of less than 0.05 was considered statistically significant.

## 3. Results

Among 1451 low- to intermediate-ASCVD-risk patients (53 ± 11 years; 51% females) with CACS <1.0 AU, 1289 (88.8%) had a CACS of zero and 162 (11.2%) had ultralow CACS (0.1–0.9 AU).

Table 1 shows the baseline characteristics of the study population before and after propensity score matching (PSM). Baseline characteristics between males and females were well balanced before PSM, except for age and body mass index, which were slightly higher in males. More females had chest pain (typical or atypical) (70% vs. 61.6%, *p* = 0.004). After PSM, the difference in chest pain symptoms was enhanced (70.8% vs. 58.4%, *p* = 0.001).

In patients with a CACS of zero, more males than females had noncalcified plaque by CTA (32.1% vs. 20.3%, *p* < 0.001). In patients with ultralow CACS 0.1–0.9 AU, the prevalence of atherosclerosis was markedly higher than in CACS 0, but not different between males and females (88% vs. 87.1%).

Table 2 shows the stenosis severity (CAD-RADS) by CTA in CACS 0 patients compared to ultralow CACS (0.1–0.9 AU) between males and females before PSM. Table 3 shows these findings after PSM. A difference in CAD-RADS stenosis severity in CACS 0 patients could be observed before and after PSM (both *p* < 0.001), but not in ultralow CACS patients (*p* = 0.106 and *p* = 0.060, respectively).

The rate of obstructive CAD (>50% stenosis, CAD-RADS 3–4) in CACS 0 patients was low and similar (6.3% vs. 4.1%, *p* = 0.11) among both sexes, while males had a higher rate of nonobstructive CAD (<50% stenosis) (25.9% vs. 16.2%; *p* < 0.001).

In ultralow CACS patients, the prevalence of obstructive CAD was higher in females (20.9% vs. 14%) compared with males, albeit not significant (*p* = 0.35). Males had more nonobstructive CAD compared with females (<50% stenosis, CAD-RADS 1 + 2) (74% vs. 66.1% *p* = 0.37).

Table 4 and Table 5 show the high-risk plaque criteria and plaque burden between males and females before and after PSM, respectively. Before PSM, all HRP criteria were found more often in males, except for spotty calcification, where a trend (*p* = 0.079) was noted. Total noncalcified plaque burden was significantly higher in males compared with females (*p* < 0.001).

**Figure 1:**
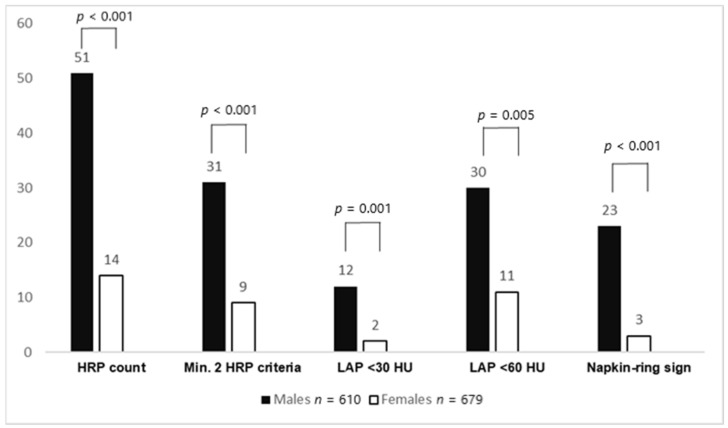
High-risk plaque (HRP) prevalence between males and females with a CACS of 0. Males had significantly more HRPs and HRP subcriteria than females. CACS: coronary artery calcium score; HRP: high-risk plaque, LAP: low-attenuation plaque.

**Figure 2:**
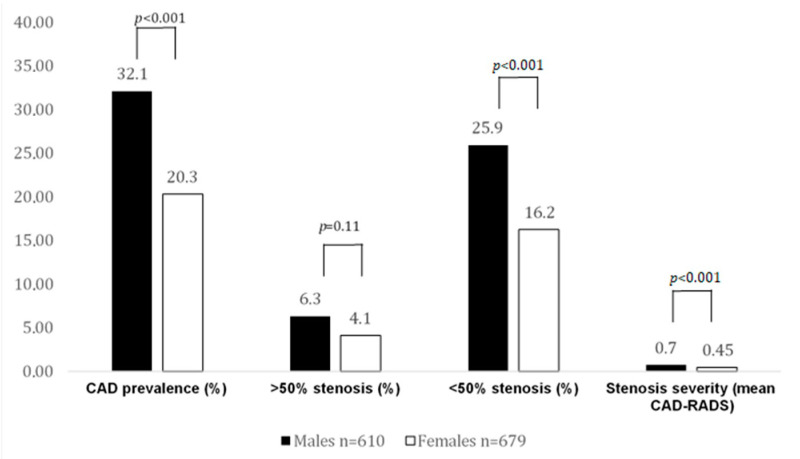
CAD prevalence and stenosis severity by CTA in CACS 0 patients. In this case, 32% of males had atherosclerosis, but significantly fewer females. Obstructive CAD rate was low and not different among sexes. More males (25.9%) with a CACS of 0 had nonobstructive disease.

Furthermore, the total number of HRPs (51 vs. 14, *p* < 0.001) was higher in males. HRPs with minimal two criteria (using LAP <60 HU as the threshold) (*p* < 0.001), LAP <30 HU (*p* = 0.005), LAP <60 HU (*p* = 0.001), LAP <90 HU (*p* < 0.001), napkin-ring sign (*p* < 0.001), positive remodeling (*p* = 0.046), and spotty calcification (*p* = 0.079) were more often found in males. Total plaque burden (G-score) (2.2 ± 4.4 vs. 1.4 ± 3.7, *p* < 0.001) was higher in males. After PSM, the abovementioned HRP criteria (except for positive remodeling) and plaque burden remained significantly higher in males. In ultralow CACS patients, no differences in HRP criteria and plaque burden between males and females could be observed, both before and after propensity score matching. Figure 1 shows the HRP prevalence between males and females with a CACS of 0, while Figure 2 displays CAD prevalence and stenosis severity in this CACS group.

Primary endpoint: (Table 6): Over a mean follow-up of 6.6 ± 4.2 years, the all-cause mortality rate in CACS 0 patients was 3.5% and 1.8% (*p* = 0.023) in females and males, respectively. In ultralow CACS patients, the rate was 0% and 3% (*p* = 0.17), respectively.

Cardiovascular mortality and MACE rates in males and females were very low (0.2% vs. 0% (*p* = 0.947) and 0.3% vs. 0.6% (*p* = 0.790), respectively).

Overall, 43.1% underwent 64-slice CTA and the remaining underwent 56.9% 128-slice dual source CTA.

## 4. Discussion

Our study revealed significant differences in the coronary atherosclerosis profile by CTA in CACS zero patients. The prevalence of noncalcified plaque, total plaque burden weighted for noncalcified, and high-risk plaque features by coronary CTA were higher in males as compared to females with CACS 0.

Significantly more males (1/3) but only 1/5 of females had noncalcified fibroatheroma on CTA (Figure 2), despite a CACS of 0. In contrast, in ultralow CACS patients, the prevalence of CAD was markedly higher, albeit similar in males and females (88% vs. 87.1%, respectively).

Previous studies have shown varying rates of nonsignificant and significant CAD by CTA among men and females ranging from 7–32% [12,13,27,28], depending on the risk profile, clinical presentation, and the CT scanner generation. Noorgard et al. [29] showed lower CACS in females and a corresponding lower rate of invasive coronary angiography and revascularization, but many open questions remained.

Previous studies enrolling patients with positive CACS above >1.0 AU reported contradictory results [30,31]. Plank et al. [30] analyzed 1050 patients (1:1 propensity score-matched) and found more calcified plaques in males, while females had more mixed and noncalcified plaques. Another study observed a higher plaque burden in males compared to females, similar to our results [31], with males having a 6–7-fold higher odds ratio for increased burden of calcified and mixed calcified plaques, whereas no sex difference was observed for noncalcified plaques. The male-to-female ratio in this study was 1.8:1 [31]. However, no studies so far have analyzed gender differences in CACS zero patients, and, to the best of our knowledge, our data are the first and only.

The higher total plaque burden in males compared with females in our study, known from cohorts with mild to severe calcium load (CACS >1.0 AU) [30,31], holds true also for patients with CACS 0.

How to manage patients with ultralow CACS (0.1–0.9 AU) is another open question. We found a relatively higher plaque burden in both males and females, without differences. Overall, total noncalcified plaque burden was higher in ultralow CACS as compared to CACS zero. Total plaque burden is an important predictor for ischemia [26] and adverse outcomes (MACEs) [32].

A strength of our study is the fact that the majority of patients who enrolled into the study underwent advanced CT technology (128-slice dual source CTA) with the highest spatial and temporal resolution and optimal image quality, as compared to previous studies using 64-slice CT [14]. Image quality plays an important role in the accurate detection of noncalcified fibroatheroma and quantification of HRP features such as LAP (HU) and the napkin-ring sign. Indeed, we found a higher prevalence of HRP in those who underwent 128-dual source CT.

**Figure 3:**
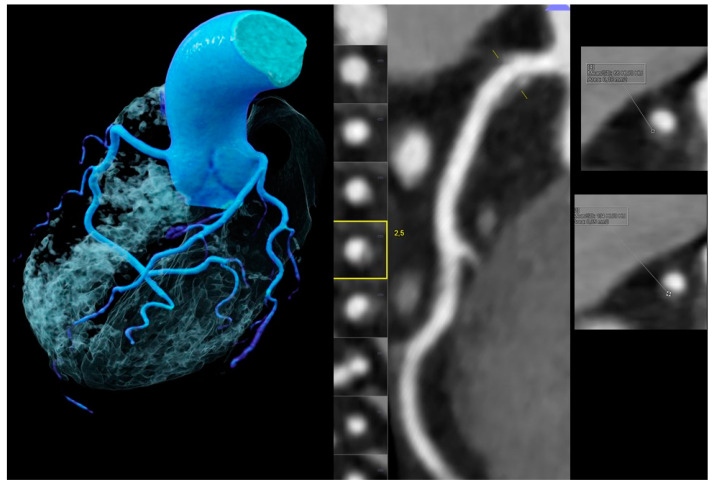
Images obtained for a 53-year-old male without chest pain but borderline ST-depression on electrocardiogram treadmill, a positive family history, and an elevated total cholesterol (234 mg/dL). Coronary artery calcium score (CACS) was zero but coronary CTA (left and mid panel, left volume rendering technique (VRT) and mid curved multiplanar reformation (MPR)) showed a mild (<50%) stenosis in the proximal right coronary artery (RCA) caused by a high-risk-plaque (HRP) with a hyperdense rim (napkin-ring sign (NRS) (*yellow square*)) (right upper and lower panels) and a very small spotty calcification (SC), which was nondetectable by CACS.

How to stratify patients with ultralow CACS is still a matter of debate. Whether ultralow CACS truly reflects coronary calcium or is merely the result of artefacts, such as noise, is not fully clear. Current standardized CT CACS scans are reconstructed with 3 mm slice thickness and may miss very small calcified nodules, while the ultrathin slices (0.75 mm) from coronary CTA allow for higher resolution and visualization [33] (Figure 3). These findings explain why “spotty calcification” nodules were found even in CACS zero patients, although the prevalence was very low (1.9% in males vs. 0.3% in females, *p* = 0.079). The other HRP features (NRS, LAP < 30, LAP < 60) were even significantly more prevalent in males. Plaques with small spotty calcification had the highest percentage of thin-cap fibroatheroma (TCFA) as compared to large spotty and dense calcifications (*p* < 0.05) in an IVUS (intravascular ultrasound) study with radiofrequency backscatter analysis; therefore, they represent important risk markers [34].

Our study is the first reporting a quantitative gender-specific analysis of HRP features in males and females with a CACS of zero. HRP criteria such as necrotic core lipid-rich low-attenuation plaque (LAP <30 HU) were linked to ischemia defined by invasive fractional flow reserve in the NXT trial (HeartFlow analysis of coronary blood flow using coronary CT angiography: NeXt sTeps) [35], and they predicted symptomatic nonobstructive lesions, as well as adverse outcomes such as MACEs [10,11]. The MACE rate was also higher for denser fibro-fatty plaque (LAP <60 HU) [11].

Recent data released from the prospective randomized Scottish COmputed Tomography of the HEART (SCOT-HEART) trial revealed that the high-risk plaque criterion LAP <30 HU (necrotic core), even at a small total plaque burden (4%), better predicts MACE than stenosis severity and the calcium score [36]. Hence, special attention must be given to the increased likelihood of HRP criteria in males with a CACS of zero, as observed in our study.

Interestingly, females were significantly more often symptomatic for chest pain, as compared to males, despite having a lower prevalence of high-risk plaque. Several factors, such as the higher prevalence of microvascular disease (cardiac syndrome X) and the smaller size of coronary arteries in females, might explain these findings [37,38,39].

Our study confirms excellent outcomes with a low all-cause mortality rate and close to zero cardiovascular mortality and MACEs in both males and females with a CACS of zero [5,6,21], which is in line with a large cohort of 66.363 patients with a CACS of zero [40].

In our population, the majority of patients with atherosclerosis by CTA despite a CACS of 0 were treated with statins after the CTA-based diagnosis of CAD, contributing to excellent outcomes.

Interestingly, females with a CACS of 0 had a significantly higher rate of all-cause mortality compared with males, but no difference in cardiovascular morality and MACEs was found. Given excellent outcomes and the overall very low mortality rates, a bias was introduced and the analysis can be regarded as underpowered. Despite age in females being slightly higher (in the unadjusted model only), it is highly unlikely that the small difference in age in our relatively “young” population was accountable for the higher mortality rate.

In summary, our study revealed significant gender differences in the atherosclerosis profile among males and females, supporting the strength of coronary CTA for early detection of CAD and coronary risk assessment [41]. Of note, our cohort represents a “real-world” population referred to coronary CTA due to low–intermediate risk, based on a previous clinical examination, but not a healthy asymptomatic screening population. Chest pain symptoms were common (2/3 were symptomatic for chest pain). In those without chest pain, CAD was suspected on the basis of other testing such as treadmill stress ECG or resting ECG, or they had a very high cardiovascular risk profile.

### Study Limitations

The majority of patients had a CACS of zero while the ultralow CACS group was very small. The major risk factors after PSM were evenly distributed. Males had a slightly higher BMI, but the difference was minimal. All-cause mortality, cardiovascular mortality, and MACE rates were very low, and outcome analyses were underpowered. The sample size of patients with ultralow CACS 0.1–0.9 was low.

## 5. Conclusions

The atherosclerosis profile of CTA in CACS zero patients showed significant gender differences. Males often had noncalcified plaques (about one-third) and nonobstructive CAD with <50% stenosis. Furthermore, males had more HRPs and a higher total plaque burden as compared to females.

Nonetheless, females had slightly worse long–term outcomes and were more frequently symptomatic for chest pain.

## Figures and Tables

**Table 1:** Baseline characteristics of the study population before (*n* = 1451) and after propensity score matching (PSM) (*n* = 742).

	Prior to PSM			After PSM +		
	Males (*n* = 710)	Females (*n* = 741)	*p*-Value	Males (*n* = 371)	Females (*n* = 371)	*p*-Value
Age (years), mean ± SD	50.5 ± 10.9	55.4 ± 10.5	<0.001 *	52.8 ± 10.6	51.2 ± 8.5	0.082 *
Age >75 years, mean ± SD	10 (1.4)	9 (1.2)	0.820	6 (1.6)	0 (0)	0.031
Body mass index (kg/m^2^), mean ± SD	26.6 ± 4	25.8 ± 5.1	<0.001 *	26.3 ± 3.8	25.6 ± 5.3	0.001 *
Body mass index >25 kg/m^2^	375 (58.3)	320 (48.2)	<0.001	217 (58.5)	185 (49.9)	0.022
Hypertension, *n (%)*	248 (42.1)	318 (51)	0.002	163 (43.9)	162 (43.7)	1.000
Current smoking, *n (%)*	217 (35.2)	180 (28.4)	0.011	121 (32.6)	133 (35.8)	0.395
Positive family history, *n (%)*	218 (37.3)	307 (51.1)	<0.001	164 (44.2)	185 (49.9)	0.141
Dyslipidemia, *n (%)*	268 (46.5)	322 (53.7)	0.017	174 (46.9)	185 (49.9)	0.463
Diabetes mellitus, *n (%)*	28 (5.1)	45 (7.8)	0.070	20 (5.4)	22 (5.9)	0.874
Symptomatic ^†^, *n (%)*	319 (61.6)	385 (70)	0.004	184 (58.4)	225 (70.8)	0.001

Parametric data are shown as the mean ± standard deviation (SD) and categorical data are shown as *n* = count (%); * Mann–Whitney test + propensity score matching (PSM) for age, body-mass index, and the five cardiovascular risk factors; ^†^ chest pain symptoms; AU = Agatston units. Positive family history is defined as myocardial infarction or sudden cardiac death in an immediate male relative <55 years or immediate female relative <65 years.

**Table 2:** Coronary computed tomography angiography (CTA) findings in patients with a coronary calcium score (CACS) of zero and with an ultralow CACS (0.1–0.9 AU) before propensity score matching (*n* = 1451); Coronary Artery Disease – Reporting and Data System (CAD-RADS)^TM^—CTA stenosis severity.

CAD-RADS^TM^	CACS 0 Males *n* = 610 *n* (%)	CACS 0 Females *n* = 679 *n* (%)	*p*-Value	CACS 0.1–0.9 Males *n* = 100 *n* (%)	CACS 0.1–0.9 Females *n* = 62 *n* (%)	*p*-Value
0	414 (67.9)	541 (79.7)	<0.001 *	12 (12)	8 (12.9)	0.106 *
1	91 (14.9)	66 (9.7)		41 (41)	32 (51.6)	
2	67 (11)	44 (6.5)		33 (33)	9 (14.5)	
3	29 (4.8)	21 (3.1)		9 (9)	10 (16.1)	
4	9 (1.5)	7 (1)		5 (5)	3 (4.8)	

* Kruskal–Wallis test; AU: Agatston units; CAD-RADS ^TM^: Coronary Artery Disease Reporting and Data System: (1) minimal <25% stenosis, (2) mild 25–49%, (3) moderate 50–69%, (4) severe ≥70% stenosis. CCS: coronary calcium score. Data are shown as *n* = count (%).

**Table 3:** Same as above after propensity score matching+ (*n* = 742); CAD-RADS^TM^—CTA Stenosis severity.

CAD-RADS^TM^	CACS 0 Males *n* = 320 *n* (%)	CACS 0 Females *n* = 328 *n* (%)	*p*-Value	CACS 0.1–0.9 Males *n* = 51 *n* (%)	CACS 0.1–0.9 Females *n* = 43 *n* (%)	*p*-Value
0	200 (62.5)	270 (82.3)	<0.001 *	6 (11.8)	6 (14)	0.060 *
1	58 (18.1)	25 (7.6)		16 (31.4)	20 (46.5)	
2	45 (14.1)	22 (6.7)		22 (43.1)	7 (16.3)	
3	11 (3.4)	7 (2.1)		4 (7.8)	8 (18.6)	
4	6 (1.9)	4 (1.2)		3 (5.9)	2 (4.7)	

* Kruskal–Wallis test + propensity score matching for age, BMI, and the five cardiovascular risk factors; AU: Agatston units; CAD-RADS ^TM^: Coronary Artery Disease Reporting and Data System: (1) minimal <25% stenosis, (2) mild 25–49%, (3) moderate 50–69%, (4) severe ≥70% stenosis. CCS: coronary calcium score. Data are shown as *n* = count (%).

**Table 4:** High-risk plaque (HRP) criteria and plaque burden by coronary CTA before propensity score matching (*n* = 1451).

	CACS 0 Males *n* = 610 *n* (%)	CACS 0 Females *n* = 679 *n* (%)	*p*-Value	CACS 0.1–0.9 Males *n* = 100 *n* (%)	CACS 0.1–0.9 Females *n* = 62 *n* (%)	*p*-Value
HRP count (*n*)	51	14	<0.001	15	5	0.227
Min. 2 HRP criteria *	31 (5.1)	9 (1.3)	<0.001	7 (7)	4 (6.5)	1.000
LAP <30 HU	12 (2)	2 (0.3)	0.005	0 (0)	2 (3.2)	0.145
LAP <60 HU	30 (4.9)	11 (1.6)	0.001	4 (4)	3 (4.8)	1.000
LAP <90 HU	36 (5.9)	13 (1.9)	<0.001	7 (7)	4 (6.5)	1.000
NRS	23 (3.8)	3 (0.4)	<0.001	7 (7)	3 (4.8)	0.743
SC	9 (1.5)	3 (0.4)	0.079	11 (11)	3 (4.8)	0.252
PR	51 (8.4)	37 (5.4)	0.046	20 (20)	11 (17.7)	0.838
Plaque burden ***	2.2 ± 4.4	1.4 ± 3.7	<0.001 **	4.2 ± 4.7	3.4 ± 5.2	0.244 **

Propensity score matching for age, BMI, and the five cardiovascular risk factors; HRP = high-risk plaque. * Minimum two criteria of four, using LAP <60 HU as the threshold. Min. = minimum; AU: Agatston units; CCS: coronary calcium score; HRP: high-risk plaque; HU: Hounsfield units; LAP: low-attenuation plaque; NRS: napkin-ring sign; PR: positive remodeling; SC: spotty calcification. Data are shown as *n* = count (%); ** Mann–Whitney U test; *** G-score.

**Table 5:** High-risk plaque (HRP) criteria and plaque burden by coronary CTA after propensity score matching (*n* = 742).

	CACS 0 Males *n* = 320 *n* (%)	CACS 0 Females *N* = 328 *n* (%)	*p*-Value	CACS 0.1–0.9 Males *n* = 51 *n* (%)	CACS 0.1–0.9 Females *n* = 43 *n* (%)	*p*-Value
HRP count (*n*)	30	5	<0.001	10	5	0.399
Min. 2 HRP criteria *	20 (6.3)	2 (0.6)	<0.001	4 (7.8)	4 (9.3)	1.000
LAP <30 HU	10 (3.1)	1 (0.3)	0.005	0 (0)	2 (4.7)	0.207
LAP <60 HU	16 (5)	2 (0.6)	0.001	2 (3.9)	3 (7)	0.657
LAP <90 HU	21 (6.6)	7 (2.1)	0.006	5 (9.8)	4 (9.3)	1.000
NRS	13 (4.1)	1 (0.3)	0.001	6 (11.8)	3 (7)	0.501
SC	6 (1.9)	1 (0.3)	0.066	7 (13.7)	3 (7)	0.336
PR	30 (9.4)	18 (5.5)	0.071	9 (17.6)	9 (20.9)	0.794
Plaque burden ***	2.6 ± 4.5	1.2 ± 3.4	<0.001 **	4.3 ± 4.8	3.3 ± 5.6	0.130 **

Propensity score matching for age, BMI, and the five cardiovascular risk factors; HRP = high-risk plaque. * Minimum two criteria of four, using LAP <60 HU as the threshold. Min. = minimum; AU: Agatston units; CCS: coronary calcium score; HRP: high-risk plaque; HU: Hounsfield units; LAP: low-attenuation plaque; NRS: napkin-ring sign; PR: positive remodeling; SC: spotty calcification. Data are shown as *n* = count (%); ** Mann–Whitney U test; *** G-score.

**Table 6:** All-cause mortality, CV mortality, and major cardiovascular event (MACE) rates between males and females with a CACS of zero and ultralow CCS (0.1–0.9) before propensity score matching (*n* = 1451).

	CACS 0 Males *n* = 610 *n* (%)	CACS 0 Females *n* = 679 *n* (%)	*p*-Value	CACS 0.1–0.9 Males *n* = 100 *n* (%)	CACS 0.1–0.9 Females *n* = 62 *n* (%)	*p*-Value
All-cause mortality	11 (1.8)	24 (3.5)	0.023	3 (3)	0 (0)	0.170
CV mortality	1 (0.2)	0 (0)	0.947	0 (0)	0 (0)	NA
MACE	2 (0.3)	4 (0.6)	0.790	0 (0)	0 (0)	NA

AU: Agatston units; CCS: coronary calcium score; CV: cardiovascular; MACE: major adverse cardiovascular events; NA: not applicable.

## Data Availability

Data are available upon reasonable request.
